# Generation of a CRISPR/Cas9-Based Vector Specific for Gene Manipulation in *Leishmania major*

**Published:** 2019

**Authors:** Ghodratollah SALEHI SANGANI, Vahid JAJARMI, Ali KHAMESIPOUR, Mahmoud MAHMOUDI, Abdolmajid FATA, Mehdi MOHEBALI

**Affiliations:** 1. Department of Medical Parasitology and Mycology, School of Public Health, Tehran University of Medical Sciences, Tehran, Iran; 2. Department of Medical Parasitology and Mycology, Faculty of Medicine, Mashhad University of Medical Sciences, Mashhad, Iran; 3. Department of Medical Biotechnology, School of Advanced Technologies in Medicine, Shahid Beheshti University of Medical Sciences, Tehran, Iran; 4. Cellular and Molecular Biology Research Center, Shahid Beheshti University of Medical Sciences, Tehran, Iran; 5. Centre for Research and Training in Skin Diseases and Leprosy, Tehran University of Medical Sciences, Tehran, Iran; 6. Department of Epidemiology and Biostatistics, School of Public Health, Tehran University of Medical Sciences, Tehran, Iran; 7. Cutaneous Leishmaniasis Research Center, Emam Reza Hospital, Mashhad University of Medical Sciences, Mashhad, Iran; 8. Research Center for Endemic Parasites of Iran, Tehran University of Medical Sciences, Tehran, Iran

**Keywords:** *Leishmania major*, CRISPR/Cas9, Gene manipulation

## Abstract

**Background:**

Gene manipulation strategies including gene knockout and editing are becoming more sophisticated in terms of mechanism of action, efficacy and ease of use. In classical molecular methods of gene knockout, homologous arms are designed for induction of crossing over event in double strand DNA. Recently, CRISPR/Cas9 system has been emerged as a precise and powerful tool for gene targeting. In this effort, we aimed to generate a CRISPR/Cas9-based vector specific for targeting genes in *Leishmania* parasites.

**Methods:**

U6 and DHFR promoters and neomycin-resistance gene were amplified from genome of *L. major* (MHRO/IR/75/ER) and pEGFP-N1, respectively. U6 promoter was cloned in pX330 vector which is named as pX330-U6. DHFR promoter and neo resistance gene sequence fragments were fused using a combination of SOE (Splicing by overlap extension)-PCR and T/A cloning techniques. To generate pX-leish, fused fragments su-bcloned into the pX330-U6. Two sgRNAs were designed to target the *gp63* gene and cloned in pX-leish.

**Results:**

The pX-leish vector was designed for simultaneous expression of cas9 and G418 resistance proteins along with a self-cleaving 2A peptide for efficient separation of the two proteins. In this study pX-leish was designed with 3 features: 1) Compatible promoters with *Leishmania* parasites. 2) Insertion of antibiotic selection marker 3) Designing an all-in-one vector containing all components required for CRISPR/Cas9 system.

**Conclusion:**

This modified system would be valuable in genome manipulation studies in *Leishmania* for vaccine research in future.

## Introduction

Leishmaniasis is a disease caused by intracellular obligate parasites with clinical manifestation from self-healing skin sores as found in cutaneous Leishmaniasis to life threatening form related to visceral Leishmaniasis (1). Chemotherapy currently is the main control strategy for Leishmaniasis, but toxic side effects and increasing drug resistance lead to failure of control measures (2). Hence development of a vaccine against Leishmaniasis has considered by researchers in recent years. Different types of vaccines have been used to protect against *Leishmania* in the past. Inoculation with live promastigotes so called leishmanization (3), killed promastigotes (4), non-pathogen *Leishmania* (5), recombinant immunogenic antigens of *Leishmania* (6), DNA vaccines (7) and various attenuated parasites (8) have been tested for their immunogenicity and protective efficacy (9). Genetically attenuated parasites and targeted elimination of virulent genes have proven to confer good protection in animal models and could be promising to find effective vaccines (10, 11).

Gene targeting is a process in which different strategies may be applied to alter or eliminate the function of a gene. These modifications can be obtained through deletion, insertion or replacement of endogenous sequence with alternative sequences (12). Homologous recombination is one of the main pathways for DNA repairing which is utilized for targeted gene replacement to generate gene knockout parasites (13).

During recent years, generation of targeted mutations has been facilitated by new gene editing systems. These systems specifically are able to induce DNA double-strand breaks (DSBs) at a target nucleotide sequence by engineered nucleases and subsequently cell repair system is activated such as CRISPR (Clustered regularly interspaced short palindromic repeats)-Cas9 system (14). The CRISPR/Cas9 system is an adaptive immune system in prokaryotes that confers resistance to viruses and other foreign genetic elements. Targeted gene disruption is created by cas9 protein directed by guide RNA (gRNA). The guide RNA sequence is complementary to the target gene sequence which is located upstream of a triple nucleotide NGG known as the proto spacer adjacent motif (PAM). Cas9 nuclease creates a double strand break three nucleotides upstream of the PAM site. In mammalian cells, DSBs are generally repaired through the Nonhomologous end joining (NHEJ) leading to small deletions and insertions (Indels) (12). But *leishmania* parasites exploit different strategy called micro-homology mediated end joining (MMEJ) to repair DSBs (15).

Recently the CRISPR/Cas9 system has been introduced as a therapeutic technology for treating genetic disorders and cancers, furthermore it is considered as a promising approach for developing effective vaccines against infectious diseases such as leishmaniasis (15). The CRISPR/Cas9 system is an appropriate and powerful tool which speed our studies up in this way. But it requires to improve the CRISPR/Cas9 system for *Leishmania* studies. Generally, the transcription of the sgRNA is under the control of U6 promoter which is recognized by RNA polymerase III, however studies have indicated that such RNA polymerase has not been characterized in trypanosomes (15, 16). Since the original vector does not possesses a *Leishmania*-driven promoter, we intended to replace the promoter with a *Leishmania*-originated promoter. On the other hand, we intend to improve pX330 vector by insertion of drug selection marker under *Leishmania*-specific promoter region.

Several Leishmanial antigens and proteins such as *gp63, LmsTI1, LeIF, TSA, P27, A2, HSPs,* Centrin and etc. play an important role in virulence of *Leishmania*. Generation of different null-mutant parasites will be possible by developed system in this study and can be studied as potential vaccine candidates. Also the identification of the novel therapeutic targets will be more feasible and efficient compared to conventional methods.

## Material and Methods

### Preparation of parasites and DNA extraction

*L. major* Promastigotes MHRO/IR/75/ER strain were grown at 22±1 °C in RPMI 1640 medium (Gibco®, BRL) supplemented with 10% heat inactivated fetal calf serum, and 100U/ml of penicillin and 100 μg/ml of streptomycin. DNA was extracted from promastigotes by phenol chloroform method as previously described (17).

### Plasmid construction

#### a) Digestion-free preparation of PCR products and cloning

The 262-bp fragment of *L. major* U6 promoter was amplified from *L. major* genomic DNA by two parallel PCRs with two sets of primers U6LmF1/U6LmR1 (reaction 1) and U6LmF2/U6LmR2 (reaction 2). Primers were designed so that BpiI restriction site sequences were bearing by primers U6LmR1 and U6LmR2, also sticky ends compatible with BpiI restriction enzyme embedded in the 5-ends of primers U6LmR1 and U6LmF2. Equimolar amounts of two PCR products (reaction 1 and 2) were mixed and heated to 95 °C for 5 min, after that was allowed to cool down to room temperature for few minutes. Reannealed DNA fragments were cloned into the BpiI site of pX330 plasmid and the resulting plasmid was called pX330-U6. All primers used are listed in Table 1.

**Table 1:** The sequence of primers used in the study

***DHFR-F***	***AATAAACCGGTGTCGACGGGGTGATGGAGAG***
DHFR-R	CCATCTTGTTCAATCATCTTCGTAGTGCTCGGACCC
Neo-F	CCGAGCACTACGAAGATGATTGAACAAGATGGATTGCACG
Neo-R1	GCGAGCTTCAGCAGATCAAAATTGAGGGTTTGCTTCACACCGGAACCGAA-GAACTCGTCAAGAAGGCGATAG
Neo-R2	TTATTACCGGTAGGACCAGGGTTGCTTTCCACGTCACCCGCGAGCTTCAG-CAGATCAAAATT
U6LmF1	TCGTCTCCATGTGAGGATTTCGTCCC
U6LmR1	AAACAGGTCTTCTCGAAGACGCACTGCAGAGAAACACGACAAAACAAACCAG
U6LmF2	CACCTCGTCTCCATGTGAGGATTTCGTCCC
U6LmR2	AGGTCTTCTCGAAGACGCACTGCAGAGAAACACGACAAAACAAACCAG
hU6F	GAGGGCCTATTTCCCATGATTCC
F- *gp63*-first	CAGTTGGACAGCAGCAGCACGCAC
R- *gp63*-first	AAACGTGCGTGCTGCTGCTGTCCA
F- *gp63*-sec	CAGTGCATCGCGTCGTGGACGCAG
R- *gp63*-sec	AAACCTGCGTCCACGACGCGATGC
F-*gp63*-up	CTCTCCCTCCCCTCGCAC
R-*gp63*-End	GGCGACGTACATCACGAAGTCG

#### b) Fusion of the DHFR promoter with Neomycine resistance gene

I. The 974-bp fragment of upstream of the DHFR gene of *L. major* was amplified by PCR with primers DHFR-F and DHFR-R.
II. The neomycin resistance gene sequence was obtained from pEGFP-N1 by PCR reaction with primers Neo-F/Neo-R1 and subsequently the 2A peptide sequence of *Leishmania* was added to the construct by latter PCR with primers Neo-F/Neo-R2. (Neo-R1 and Neo-R2 are long primers contain 2A peptide sequence of *Leishmania*).
III The 974-bp fragment of regulatory region of DHFR gene from step “i” and 883-bp fragment of neomycin resistance gene sequence followed by the 2A sequence of *Leishmania* from step “ii” were fused together by SOEPCR with primer pair DHFR-F and Neo-R2 containing AgeI enzyme restriction site.

#### c) Cloning of the construct

SOE-PCR product (DHFR -Neo-2A fragment) was first ligated into the linearized pTG19 vector (T/A cloning). We named this vector as pTG19-DHFR-Neo.

**Fig. 1:**
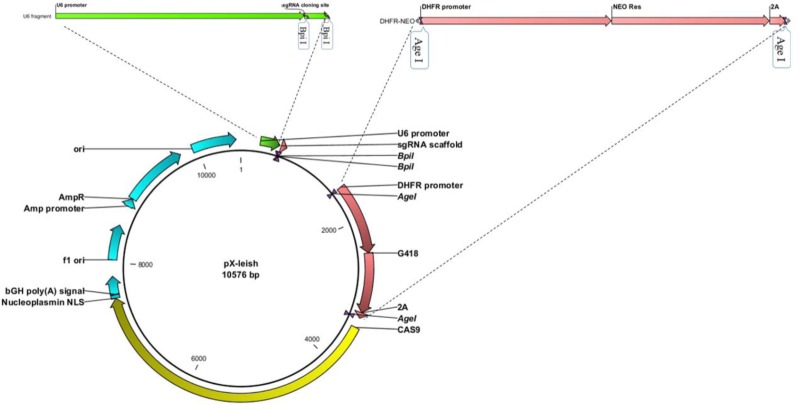
pX-leish: U6 promoter shown in green and DHFR promoter and Neo resistance gene sequence in red

Then pTG19- DHFR-Neo was digested by Age I restriction enzyme. The 1825-bp fragment was extracted from agarose gel by NucleoSpin® Gel and PCR Clean-up kit (MACHEREY-NAGEL GmbH, Germany) and cloned into Age I restriction site of pX330-U6. The resulting plasmid was named pX-leish (Fig. 1).

### Preparation of the vector containing sgRNAs

#### Designing of sgRNAs

Two guide RNAs were designed for *gp63* gene of *L. major* on plus and minus strand. Complementary oligonucleotides of each guide RNA sequence were first mixed at equimolar concentration and annealed by heating at 95 °C for 5 min and then shaked gently and immediately cooled down and incubated on ice for few minutes. The annealed oligonucleotides were then cloned into BpiI restriction sites of pX-leish.

Human U6 promoter is a member of the RNA polymerase III promoters that has been used to drive gRNA transcription in mammalian cells. U6 snRNA regulatory elements have been already utilized for transcription of the sgRNA in *P. falciparum* and *L. major* (18, 19).

### Leishmania transfection and antibiotic selection

*Leishmania* transfection was performed by electroporation method by an electroporation system (Eppendorf® Multiporator® 36205-10) at 2500V and 5 ms. An electroporation reaction was also subjected as control in the same conditions without addition of DNA. G418 treatment was started with concentration of 15 μg/ml and increased up to 50μg/ml in the next days.

### Evaluation of the pX-Leish vector efficiency

Genomic DNA was extracted from electroporated parasites and wild parasites and utilized as the template for amplification of an 800bp fragment containing the target sequences of sgRNA and PAM for targeting by Cas9 protein. Disruption of *gp63* gene was evaluated through the comparison of amplified fragment in transfected and wild type parasites. Also purified PCR products of transfected and WT parasites were mixed well together and denatured at 95 °C for 10 min. Then cooled down to room temperature. Digestion of annealed PCR products was carried out by T7 endonuclease I for 1 hour at 37 °C. T7 Endonuclease I was inactivated using proteinase K and incubation at 37 °C for 10 min. Analysis of experiment was performed by gel electrophoresis of digestion products.

## Results

### Modification of U6 promoter

**Fig. 2:**
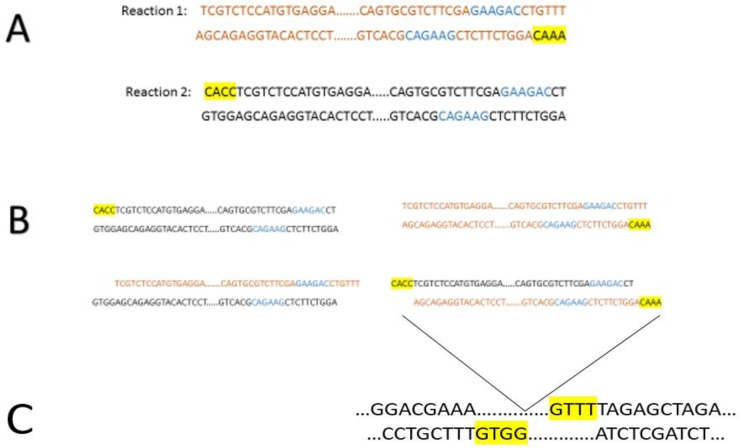
Preparation of U6 snRNA promoter, A) PCR products of reaction 1 and 2. B) Re-annealing fragments indicates that 25% of them bearing sticky ends of interest. (BpiI enzyme restriction sites are shown colored font in blue) C). Bpi I restriction site on the pX330

**Fig. 3:**
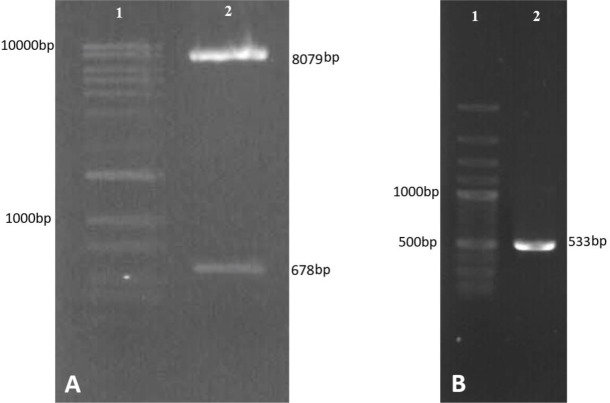
Confirmation of U6 cloning. A)Lane 1; DNA marker, Lane 2;digestion of pX330-u6 by Ndei B) Lane 1; DNA marker, Lane 2; PCR product of U6 promoter using primers hU6 and U6LmR2 in the reaction

For sgRNA transcription, we also used the upstream sequences of U6 snRNA on chromosome 24 of *L. major* (KEGG T01014: LMJF24_snRNA_01). Amplification of 262-bp sequences of upstream region of U6 snRNA by two parallel PCR reactions and mixing of their products (as described above) to generate sticky ends required for ligation in 25% of re-annealed fragments. As shown in Fig. 2, resulting sticky ends are complementary to Bpi I restriction site on the pX330. Cloning was confirmed by amplification of the insert (by primers hU6F and U6LmR2) and enzyme digestion of the plasmid. Digestion by NdeI restriction enzyme produced two fragments corresponding in size to the 8079bp and 678 bp bands (Fig. 3).

### Preparation of spliced fragments

**Fig. 4:**
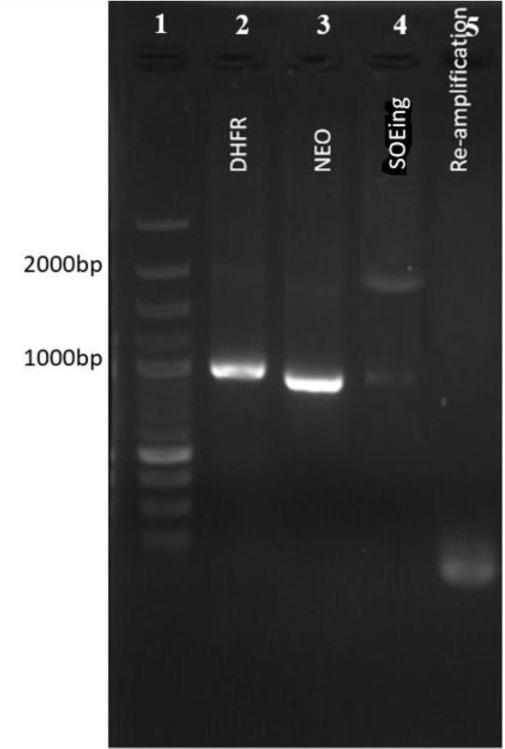
SOEing PCR. Lane1; DNA marker, Lane2; DHFR promoter fragment, Lane3; Neo gene fragment, Lane4; Unsuccessful SOE-PCR. Lane5; Re-amplification from SOEing PCR product

Fusion of the two fragments regulatory region of the DHFR gene and the neomycin resistance of SOE-PCR product displayed an expected band in size, but did not show a clear and single band and adequate intensity as well. Subsequently, a PCR reaction was performed using the SOE-PCR product but no bands observed following gel electrophoresis (Fig. 4).

**Fig. 5:**
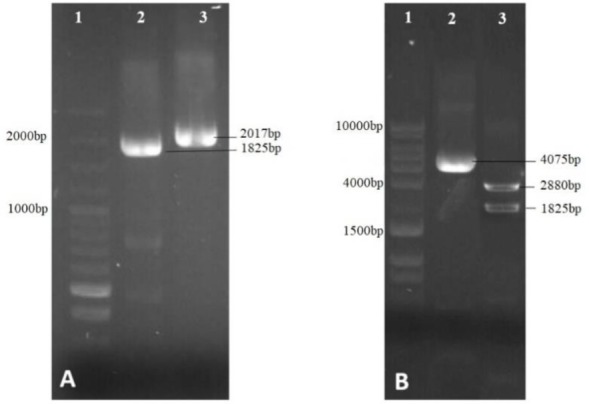
T/A cloning confirmation. A) Lane1; DNA marker, Lane 2; PCR reaction using specific primers, Lane 3; PCR reaction using M13 universal primers. B) Lane1; DNA marker, Lane 2; pTG19-DHFR-Neo, Lane 3; Age I digestion

In an attempt to alleviate the problem following a SOE-PCR reaction, the construct (SOEPCR product) was cloned into a T-vector (linearized pTG19). T/A cloning was confirmed by PCR experiments using M13 and specific primers as forward and reverse primers, respectively (Fig. 5). Amplification of SOE construct was successfully performed from pTG19-DHFR-Neo. SOE construct was obtained in high yield in this way and successfully sub-cloned into pX330-U6.

### Cloning of the SOE-PCR product

**Fig. 6:**
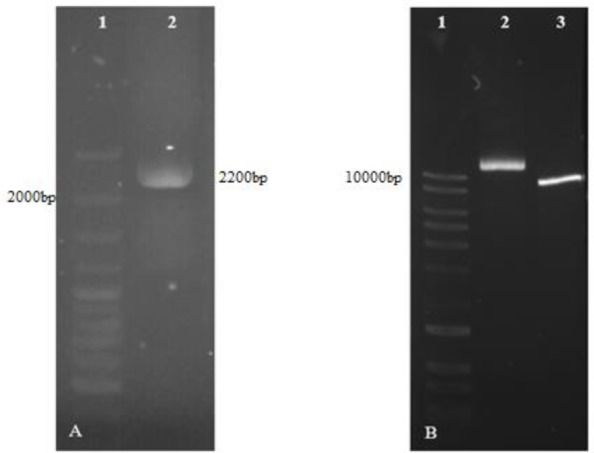
Confirmation of sub-cloning. A) Lane1; DNA marker, Lane 2; PCR reaction using primers U6LmF1/DHFR-R B) Lane1; DNA marker, Lane 2; pX-leish digestion by XhoI Lane 3; circular pX-leish

The insert (construct) for ligation was prepared in high yield with enzymatic digestion of pTG19-DHFR-Neo by Age I that showed the 1825-bp DNA fragment (Fig. 5). Colony PCR results showed successful cloning of SOE-construct into pX330-U6. Resulting plasmid was described as pX-leish. Cloning was confirmed by amplification of SOE-construct from extracted pX-leish and enzymatic digestion. Moreover correct orientation of construct was confirmed by amplification of a 2200-bp fragment using primers U6LmF1/DHFR-R. pX-leish has a XhoI restriction site on the SOE construct sequence but not on the pX330-U6. pX-leish was linearized by XhoI restriction enzyme (Fig. 6).

### Cloning of specific guide RNAs for gp63

**Fig. 7:**
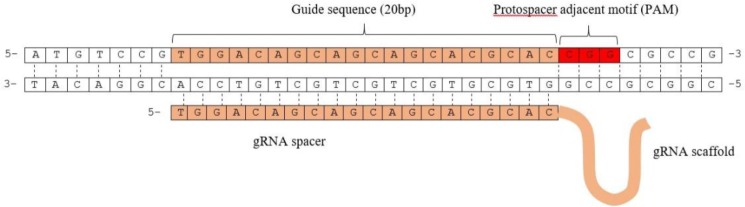
Schematic representation of the Protospacer and Pam sequence located on *gp63* Gene

**Fig. 8:**
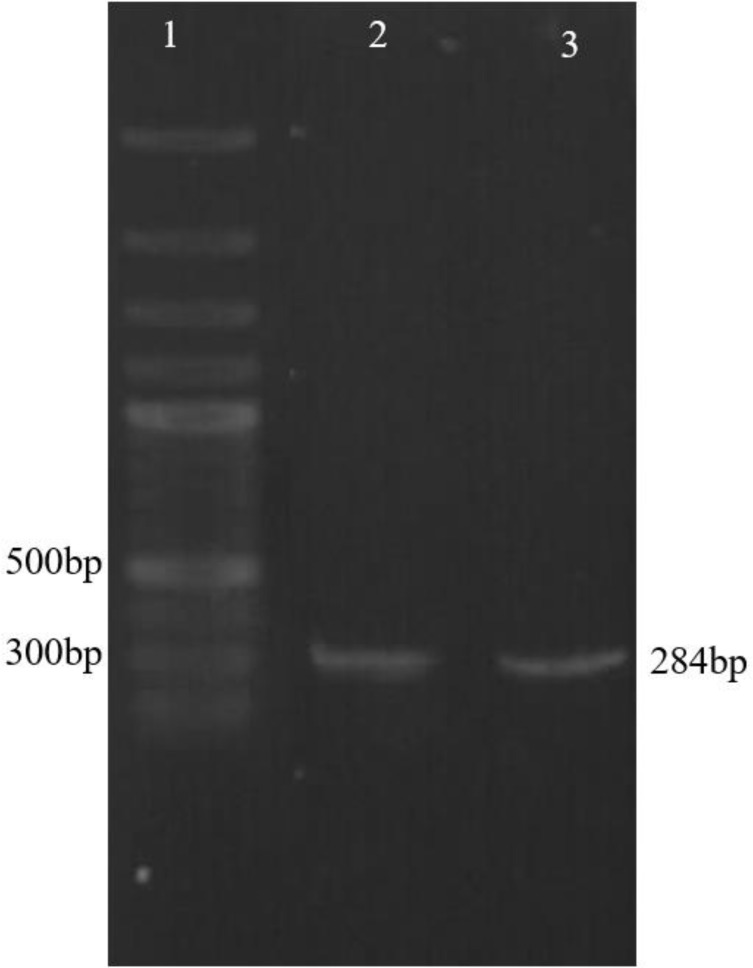
Confirmation of sgRNA cloning. Lane1; DNA marker, Lane 2; PCR reaction using U6LmF1 primer and sgRNA (R-*gp63*-first) as reverse primer, Lane 3; PCR reaction using U6LmF1 primer and sgRNA (R-*gp63*-second) as reverse primer

Following the preparation of the vector containing a neomycin resistance gene fused to Cas9 under the regulatory elements of the DHFR gene, the two sgRNAs for targeting the *gp63* Gene were cloned into the site of BpiI restriction enzyme downstream of the U6 promoter in the vector (Fig. 7). Amplification of U6 fragment using a strand of sgRNA sequence as reverse primer demonstrated the accuracy of cloning of sgRNAs (Fig. 8).

### Assessment of double-strand DNA breaks (DSBs)

**Fig. 9:**
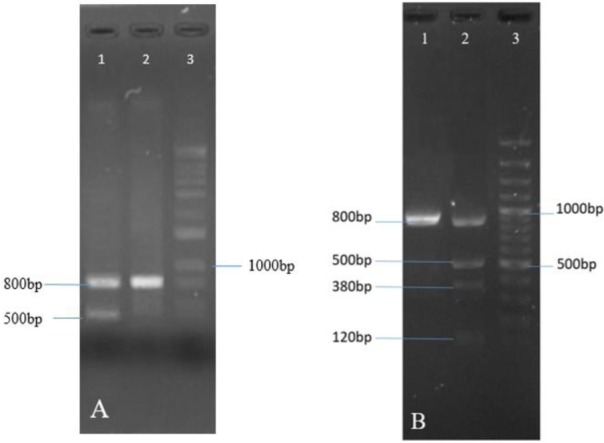
Confirmation of pX-Leish efficiency. A) Agarose Gel detection of PCR amplicon resulted from MMEJ. Lanes 1 and 2; PCR products of transfected and WT parasites by primers F*gp63-*up/R*gp63*-end respectively; Lane 3, DNA Marker; B) Digestion of PCR products by T7 Endonuclease: Lane 1; Digestion of PCR product of DNA extracted from WT parasites; Lane 2, Digestion of mixed PCR products of WT and transfected parsites; Lane 3, DNA marker

To evaluate the capability of the vector to target the gene of interest at the regions specific for guide RNAs, the *L. major* parasites were transfected with pX-Leish and incubated at 22 °C for 24 hours. Then the culture medium was replaced with RPMI 1640 containing 15 μg/ml of G418. Unlike what we expected, after electroporation and allowing parasites to grow, the transfected cell growth was arrested. Different protocols were applied for transfection with no success in survival. Comparison of the amplified fragments from extracted DNA of transfected and WT parasites verified the pX-Leish efficiency. The expected size of amplified fragment was 800bp as observed in PCR from DNA of WT parasites, whereas amplified fragment from DNA of transfected parasites was 500bp (Fig. 9). Furthermore, deletion or insertion was evaluated by digestion of the re-annealed heterogenic PCR products with T7 Endonuclease I. Gel analysis indicated multiple bands corresponding to detected fragments about 800 bp, 500 bp, 400 bp and 100 bp sizes (Fig. 9).

The 800 bp length is original size related to WT parasites. The 400 bp- and 100 bp- fragments resulted from digestion of 500 bp fragment in size that confirm the deletion occurred in target sites.

In a different experiment we allowed to growth electroporated parasites in an antibiotic free RPMI medium, passaged in a fresh RPMI in a ratio of 1:10, gradually a population of promastigotes grew up during 2 weeks. Amplification of 800 bp-fragment was performed to evaluate mutation of *gp63* gene in this population. As expected, we observed the same electrophoresis pattern in PCR amplicon of WT parasites. This indicates transfected promastigotes could not growth in the antibiotic free medium and therefore new populations were untransfected promastigotes.

## Discussion

Until recently, homologous recombination has been one of the main techniques in genetic manipulation of *Leishmania*. Generation of knockout parasites has been common method via targeted gene replacement with antibiotic selection marker.

Although tolerance of homologous recombination by *Leishmania* is considered as an advantageous trait for generation of knockout parasites, but this method is labor, time consuming with low rate of recombination (15).

Over the last few years, a more efficient genome editing technology known as CRISPR/Cas9 system has been used in mammalian cells and other organisms such as protozoan parasites. This method has improved our ability in genome editing of *Leishmania*. The current available vectors are designed for mostly mammalian cells which means that if the usage of such system in other eukaryotic cells is required, the replacement of promoters would be necessary. We constructed a CRISPR/Cas9 system compatible with *Leishmania* containing antibiotic selection marker. For this end, we modified Px330 vector and constructed an all-in-one vector containing single guide RNA expression cassette and Neo-Cas9 expression cassette.

For transcription of the sgRNA, U6 small nuclear RNA (snRNA) regulatory elements have been already used in *P. falciparum* and *L. major*(18, 19). RNA polymerase I promoter was also implemented in CRISPR/Cas9 system for expression of gRNA in *L. donovani* (15). Valerian Nakaar and his team demonstrated that two intragenic regulatory elements of tRNA genes namely A and B boxes act as extragenic regulatory elements for U6 snRNA gene in Trypanosomes. They discovered A and B boxes at upstream of U6 snRNA gene are essential for expression of U6 snRNA gene and deletion of these boxes resulted in undetectable levels of the U6 snRNA gene expression (20). We found U6 snRNA sequences of *L. major* on the minus strand of chromosome 24 using BLAST alignment of U6snRNA sequences of *T. brucei*. Therefore we implemented a 262-bp sequence of 5′-upstream region of the transcriptional start site of U6 snRNA as the sgRNA promoter. On the other hand, we re-established the BpiI enzyme restriction site by addition of the 12-bp fragment at the 5′-end of reverse primers (Fig. 1). The designed sgRNAs in this study were cloned successfully in to the pX-leish.

Current vectors are used for targeting the mammalian genomes. Different promoters have been applied for expression of cas9 gene in parasites such as: the alpha-tubulin promoter in *Toxoplasma gondi*(21), plasmodial regulatory elements in *Plasmodium falciparum*(18), *Leishmania* tubulin intergeneic region in *L. donovani* (15), DHFR-TS promoter in *L. major* (19) and ribosomal promoter in *Trypanosoma cruzi* (22).

Dihydrofolate reductase (DHFR) is an essential enzyme was express in the both promastigote and amastigote forms of *Leishmania* (23). Hence we employed 5′-flanking region of DHFR gene of L. major for expression of antibiotic resistance marker and cas9 protein. In order to simultaneous expression of Cas9 and antibiotic resistance proteins a self-cleaving 2A peptide was used to mediate efficiently co-translational “cleavage” between the upstream of cas9 and downstream of Neo gene. The 2A self-cleaving peptide allows them to be encoded as a polyprotein and dissociate from each other during translation. Function of 2A peptide has already proved in various parasites such as *Eimeria tenella, Plasmodium yoelii, Trypanosoma cruzi* (24–27).

In mammalian cells, DSBs are repaired through either introducing homologous DNA sequences (homologous recombination) or NHEJ. High rates of DSBs increases the frequency of homologous recombination in presence of the homologous template. Also repair of DNA occurs by NHEJ when homologous template is absent. Small deletions and insertions (Indels) occurred during NHEJ may lead to mutation at the target site with subsequent alteration or disruption of protein function (12). Observations has been indicated that NHEJ is absent in *Leishmania*, but it has been demonstrated that *L. donovani* utilizes MMEJ to repair the DSB-induced by Cas9-nuclease(15). Distance of microhomology sequences to the DSB site determines the length of deletion (15, 28).

In the current study selected PAMs were located near the 5′end of the gp63 coding sequence close to the start codon. The guide RNAs were designed against sequences upstream of the PAM regions. T7 endonuclease digestion proved a deletion in the region of the PAM sequence. This assay was performed as a primary confirmation of the CRISPR/cas9 activity. However western blotting would confirm the absence of the protein.

The gp63 sequence is highly GC-rich especially at the 5′-end where designed sgRNAs. Therefore it make difficult to design high specific sgRNAs for this region. In current study we tried to select sgRNAs with high score. But recently more powerful softwares are available to recognize sequences with least toxicity. Previous studies described GC-rich sequences may yield more off-targets (29). Given that efficacy of pX-Leish, to understand the reason which we not able to obtain mutant live parasites, we speculate that designed sgRNAs probably have been non-specific for gp63 gene and generated the various off targets on the *Leishmania* genome and induced apoptosis and death of parasites. For this purpose we evaluated sgRNAs again with online special softwares and they were not ranked with high score. Although we cannot certainly assert that off targets have been the arrest cause of parasites, but it is a strong possibility and only reason we speculate that may cause multiple DNA fragmentation and death.

## Conclusion

The pX-leish can be utilized for gene targeting of any virulent antigens in *Leishmania* or even it may be possible to design sgRNAs or targeting different genes simultaneously. The CRISPR/cas9 technique benefits from this fact that it is capable of targeting gene of interest simply by designing specific sgRNAs followed by an easy method for cloning the fragments into the vector. Ease and high efficiency potential of this method may help for example generation of attenuated parasites as a vaccine, identification of therapeutic targets and function of genes. The current study was conducted to improve this system which may able to open promising ways for control measures of leishmaniasis.
